# Measles immunity gaps among children and adolescents with HIV in Zambia despite high measles vaccination and antiretroviral therapy coverage

**DOI:** 10.1097/QAD.0000000000003634

**Published:** 2023-06-28

**Authors:** Simon Mutembo, Yangyupei Yang, Andrea Carcelen, Amy Kaye Winter, Francis Dien Mwansa, Innocent Chilumba, Irene Mutale, Gershom Chongwe, Mwaka Monze, Gina Mulundu, Hope Nkamba, Lloyd Mulenga, Kyla Hayford, William John Moss

**Affiliations:** a Department of International Health, International Vaccine Access Center, Johns Hopkins Bloomberg School of Public Health, Baltimore, Maryland, USA; b Ministry of Health, Government of the Republic of Zambia, Lusaka, Zambia; c Department of Epidemiology and Biostatistics, University of Georgia, Athens, Georgia, USA; d Tropical Diseases Research Center, Ndola; e Department of Pathology and Microbiology, University Teaching Hospital; f Ministry of Health, Directorate of Clinical Care and Diagnostic Service, Government of the Republic of Zambia, Lusaka, Zambia; g Department of Epidemiology; h W Harry Feinstone Department of Molecular Microbiology and Immunology, Johns Hopkins Bloomberg School of Public Health, Baltimore, Maryland, USA.

**Keywords:** biorepository, HIV, measles, rubella, seroprevalence, serosurvey

## Abstract

**Objective:**

The study objective was to identify measles and rubella immunity gaps among people with HIV (PWH) in Zambia despite high measles vaccine coverage and widespread access to antiretroviral therapy.

**Design:**

Nationally representative cross-sectional serosurvey using biorepository specimens.

**Methods:**

Blood specimens collected in the Zambia Population HIV Impact Assessment survey (ZAMPHIA) of 2016 were tested for measles and rubella immunoglobulin G (IgG) antibodies by enzyme immunoassay. Hierarchical generalized additive models were fit to characterize age-specific measles and rubella seroprevalence profiles by HIV infection status. Log-binomial regression was performed to identify factors associated with seronegativity.

**Results:**

Of the 25 383 specimens, a subsample of 11 500 were selected and 9852 (85%) were successfully tested. Measles seroprevalence was lower among PWH compared with HIV-uninfected individuals until approximately 30 years of age. Among children younger than the age of 10 years, measles seroprevalence was 47.2% [95% confidence interval (CI): 32.7, 61.7] in PWH and 76.4% (95% CI: 74.9, 78.0) in HIV-uninfected children in same age category. In contrast, rubella seroprevalence was higher among PWH than HIV-uninfected individuals, particularly for children younger than 10 years (68.6% vs. 44.3%, *P* < 0.001). Having a detectable viral load was associated with being measles seronegative (adjusted prevalence ratio 0.15, 95% CI: 0.06, 0.38).

**Conclusions:**

These results from a nationally representative serosurvey demonstrate persistence of measles immunity gaps among PWH younger than 30 years of age. There is need to implement the World Health Organization's recommendation to revaccinate children living with HIV against measles following immune reconstitution with antiretroviral therapy to protect these children and prevent measles outbreaks.

## Introduction

Measles and rubella remain important causes of morbidity and mortality. In 2021, there were an estimated 9.5 million measles cases and 128 000 measles deaths globally [1]. Although the number of measles cases and deaths decreased during the coronavirus disease 2019 (COVID-19) pandemic [2], measles is again resurging likely due in part to pandemic disruptions to routine immunization and mass vaccination campaigns [3,4]. Rubella results in an estimated 100 000 annual deaths globally due to congenital rubella syndrome (CRS) [5]. Both viruses are endemic in sub-Saharan Africa and no country in the African Region has eliminated measles or rubella despite a regional measles elimination goal of 2020 [6].

High levels of population immunity are needed to interrupt measles virus transmission and even small clusters of susceptible individuals can sustain measles outbreaks [7]. Although the most important obstacle to measles control and elimination is low vaccination coverage with two doses of a measles-containing vaccine (MCV) [8], high numbers of children living with HIV could result in more susceptible children and enhance the risk of measles outbreaks [9]. Reasons for increased measles susceptibility among children living with HIV are several. Children born to HIV-infected mothers have lower levels of passively acquired maternal antibodies, increasing susceptibility to measles at a younger age [10]. Children with HIV may have a poor primary response to measles vaccine and protective antibody levels following vaccination may wane within several years in children not receiving antiretroviral therapy [11]. Highly active antiretroviral therapy does not restore measles immunity when started after measles vaccination [12], although children vaccinated against measles while receiving antiretroviral therapy have a more durable antibody response [13]. As a consequence, the World Health Organization recommends that children living with HIV be vaccinated against measles at 6 months rather than 9 months of age and be revaccinated against measles following immune reconstitution with combination antiretroviral therapy [14]. However, these recommendations are rarely implemented in many countries.

Several systematic reviews found that measles seropositivity was lower among vaccinated children living with HIV compared with HIV-uninfected children [15–17], consistent with higher rates of primary and secondary measles vaccine failure among children living with HIV. In contrast, a systematic review of measles seroprevalence among adolescents and adults living with HIV found no differences in measles seropositivity compared with HIV-uninfected individuals [18]. These differences in measles seropositivity by age among PWH reflect differences in the relative timing of HIV infection and exposure to measles vaccine or wild-type virus. In children, exposure to measles vaccine or virus most commonly occurs in the context of an impaired immune system following perinatal HIV infection whereas in adults HIV infection typically occurs after immunity to measles virus is established [19].

The studies summarized in the systematic reviews, however, were relatively small, highly heterogenous in study design and laboratory methods, and conducted in specific study populations [20]. No nationally representative, population level studies of measles and rubella seroprevalence have been reported among children and adults with HIV, particularly in a setting with high measles vaccine coverage and access to highly active antiretroviral therapy. Zambia has high coverage with the first dose of measles-containing vaccine (MCV) (≥90% since 2015) [21], increasing coverage with the second dose of MCV (81% in 2021) [21], and access to highly active antiretroviral therapy. In 2020, 81% of HIV-positive adults received antiretroviral therapy [22] and, in 2019, pediatric antiretroviral coverage was 83% and viral suppression was 72% among children younger than 15 years who had viral load test results [22].

This is the first report of measles and rubella seroprevalence by HIV infection status based on a nationally representative sample and demonstrates persistence of measles immunity gaps among children, adolescents and young adults living with HIV.

## Methods

### ZAMPHIA study design and laboratory procedures

A nationally representative, cross-sectional measles and rubella serosurvey was conducted by analyzing a subsample of blood specimens collected in the Zambia Population HIV Impact Assessment survey (ZAMPHIA) [23]. ZAMPHIA was a nationwide, cross-sectional, household survey conducted in 2016 to estimate HIV incidence and prevalence in Zambia [24] and prior to the introduction of rubella vaccine into the routine immunization program in Zambia. This survey collected demographic information and blood specimens from adults 15 to 49 years of age in selected households and from children younger than 15 years of age from every other selected household. However, history of measles and rubella vaccination was not collected. Venous blood was collected from those older than two years of age and capillary blood was collected as dried blood spots (DBS) from children younger than 2 years. After HIV testing was completed, residual specimens were frozen at −70°C and archived at the Tropical Diseases Research Centre (TDRC) in Ndola, Zambia.

HIV testing was performed as part of the ZAMPHIA study using two HIV rapid tests, Determine and Unigold [24]. Individuals with a nonreactive result on the Determine test were classified as HIV negative. Individuals with a reactive screening test result underwent confirmatory testing using the Unigold test. Those with a reactive result on both tests were classified as HIV positive. Individuals with a reactive screening test result followed by a nonreactive confirmatory test result were classified as indeterminate and requested to have repeat testing in four weeks. Specimens from participants who self-reported being HIV positive but tested negative underwent additional HIV nucleic acid testing. For infants younger than 18 months who screened positive by rapid test, virological testing was conducted to detect HIV DNA by PCR on the Roche COBAS AmpliPrep Instrument and COBAS TaqMan 48 Analyzer using the COBAS AmpliPrep/COBAS Taqman HIV-1 Qualitative Test (Roche Molecular Diagnostics, Branchburg, New Jersey, USA) [24].

For participants identified as HIV positive, additional assays were performed as part of the ZAMPHIA study to measure CD4^+^ T-lymphocyte counts (data on CD4^+^ T-lymphocyte percentage were not available), HIV-1 viral load, antiretroviral drug use, and recency of HIV-1 infection. HIV viral load suppression was defined as an HIV viral load of less than 1000 copies/ml. Qualitative screening for detectable concentrations of antiretroviral drugs was performed using DBS from HIV-positive children and adults using high-resolution liquid chromatography and tandem mass spectrometry. Efavirenz, atazanavir and lopinavir were selected as indicators of the most prescribed first- and second-line regimens. Specimens from participants with suppressed viral loads or those who self-reported taking antiretroviral drugs but had no evidence of the three indicator drugs were tested for nevirapine. To distinguish recent from long-term HIV infection, two testing algorithms were used: HIV-1 LAg Avidity enzyme immunoassay (Sedia Biosciences 25 Corporation, Portland, Oregon, USA) with HIV viral load; and HIV-1 LAg Avidity EIA, viral load, and antiretroviral drug detection Specimens with a median normalized optical density of ≤1.5 were classified as potentially representing recent HIV infection and underwent HIV-1 viral load testing for further classification. Specimens with a viral load of <1000 copies/ml were classified as long-term infections whereas those with a viral load of ≥1000 copies/ml were classified as recent infections. In the second algorithm that adjusted for antiretroviral drug use, specimens with a viral load ≥1000 copies/ml and with detectable antiretroviral drugs were classified as long-term infections. Specimens with VL ≥1000 copies/ml and without detectable antiretroviral drugs were classified as recent infections [24]. The final determination of HIV infection status, antiretroviral drug use, and recency of HIV infection in the ZAMPHIA study was used in this analysis.

### Selection of ZAMPHIA specimens for measles and rubella serology

To compare measles and rubella seroprevalence between children and adults living with HIV and those who were HIV uninfected, a provincially representative subsample of specimens was selected from the ZAMPHIA biorepository based on HIV infection status, cluster representation, and age. All HIV-positive participants and all participants from small clusters (≤10 participants) were included to generate representative estimates. Selected specimens from the ZAMPHIA biorepository were weighted by province and age category (0–4, 5–9, 15–19, and 20–49 years), the same age categories used for weighting in the ZAMPHIA survey. Specimens for which consent for future testing was not obtained were excluded. Details of sampling and weighting are described in more detail in a separate publication [23].

### Measles and rubella serological assays

Serum was eluted from DBS collected from children younger than two years [23]. The eluted sera and plasma specimens from older children and adults were tested for IgG antibodies to measles and rubella viruses using commercial enzyme-linked immunoassays (Euroimmun Anti-Measles Virus ELISA and Euroimmun Anti-Rubella Virus ELISA, Germany). Participants were classified as measles seropositive if measles virus immunoglobulin G (IgG) antibody concentrations were greater than or equal to 200 mIU/ml, equivocal if between 150 and 200 mIU/ml, and negative if <150 mIU/ml. Similarly, participants were classified as rubella seropositive if rubella virus IgG antibody concentrations were greater than or equal to 11 IU/ml, equivocal if between 8 and 11 IU/ml, and negative if less than 8 IU/ml. Equivocal results were considered positive for these analyses.

### Statistical analysis

Hierarchical generalized additive models were fit to individual measles and rubella seropositivity to characterize age-specific measles and rubella seroprevalence profiles by HIV infection status. Hierarchical generalized additive models allowed modeling of interactions between HIV infection status and age with different smoothers. The final measles and rubella model included a global smoother over age (i.e. cyclic cubic regression splines), and group-level (i.e. HIV status level) smoothers with the shared penalty. The shared penalty influences basis function coefficients to prevent over fitting and ensure appropriate complexity of each smoother, meaning that each HIV status group has the same smoothness. The choice of the basis dimension was large enough to have sufficient degrees of freedom to represent the underlying data. The best model was selected between two link functions (logit and complementary log-log) by minimizing deviance. The final measles and rubella model included a logit link. All statistical analyses were performed in R (R Foundation for Statistical Computing, Vienna, Austria) and all hierarchical generalized additive models were fit using the ‘mgcv’ package in R [25].

Log-binomial regression was performed to assess associations between measles and rubella seroprevalence and HIV infection status and other risk factors. Prevalence ratios and corresponding 95% confidence intervals were calculated as the measure of effect. Forward and backward stepwise selection methods were used to select the best fit model. Variables with a *P*-value <0.25 from univariate analysis were included in the final multivariable model. Log likelihood and Akaike's information criteria (AIC) were used to determine the goodness of fit of the adjusted final model in comparison to the preceding models, and the model with the lowest AIC was selected as the best fit model. *Survey* package was used for descriptive results and log-binomial regression was used to account for sampling weights [26]. Figures were generated using the ggplot2 package [27].

### Ethical approval

Approval to conduct this study was obtained from the Johns Hopkins University School of Public Health Institutional Review Board and Tropical Diseases Research Center Research Ethics Committee. Further regulatory authority was obtained from the Zambia National Health Research Authority. The Ministry of Health in Zambia and the Centers for Diseases Control and prevention, Zambia country office authorized the use of the ZAMPHIA biorepository.

### Role of funding source

The funder did not have a role in study design; in the collection, analysis, and interpretation of data; or in the decision to submit the paper for publication.

## Results

### Characteristics of the study population

Of the 25 383 specimens in the ZAMPHIA biorepository, 11 500 specimens were selected for measles and rubella serological testing, representing individuals younger than 50 years of age from the 10 provinces in Zambia (Figure S1, Supplemental Digital Content). Of the selected specimens, 9852 (85%) were tested for measles and rubella IgG antibodies by enzyme immunoassay [23]. Of these, 331 (3.4%) were from PWH and 9521 (96.6%) from HIV-uninfected participants. After weighting by province and age category, the analyses included 5.6% PWH and 94.4% HIV-uninfected participants (Table 1).

**Table 1:** Characteristics of the study population by HIV infection status after weighting by province and age category.

Characteristic	Overall	HIV-uninfected	People with HIV
Unweighted number (%)	9852	9521 (96.6)	331 (3.4)
Weighted percentage		94.4	5.6
Age			
Median in years (IQR)	12 (6,18)	12 (6,18)	22 (16,36)
Sex (%)			
Male	53.1	53.3	48.3
Female	46.9	46.7	51.7
Setting (%)			
Rural	64.6	65.1	50.1
Urban	35.4	34.9	49.9
Province (%)			
Central	9.4	9.3	13.0
Copperbelt	12.2	12.1	14.5
Eastern	10.0	10.2	4.5
Luapula	8.3	8.3	7.3
Lusaka	12.2	12.1	16.3
Muchinga	11.3	11.6	3.0
Northern	8.1	8.1	7.3
North-Western	10.2	10.4	4.5
Southern	11.2	11.1	13.0
Western	7.1	6.8	16.6

Based on weighted results, the median age was 22 years [interquartile range (IQR): 16–36 years] for PWH and 12 years (IQR: 6–18 years) for HIV-uninfected participants, reflecting the small number of children living with HIV in the study population (Table 1). After weighting, a slightly higher proportion of PWH were female (51.7%) compared to HIV-uninfected participants (46.7%), and the proportion of PWH residing in urban areas (49.9%) was higher than for HIV-uninfected participants (34.9%) (Table 1).

Most (97%) PWH did not have evidence of recent HIV infection (Table 2). Of the PWH, 82.5% self-reported receiving antiretroviral therapy ART or tested positive for biomarkers of antiretroviral drugs indicating they were receiving therapy (Table 2). One-eighth (12.4%) of PWH had CD4^+^ T-lymphocyte cells counts < 200 cells/μl, indicative of severe immunosuppression, and this proportion was higher among adults (14.7%) than children younger than 15 years (5.4%) (Table 2). Of those with evidence of receiving antiretroviral therapy, 76% achieved HIV viral suppression but only 39.6% of all HIV-infected participants achieved viral suppression, a proportion that was higher among adults (49.2%) than children younger than 15 years (35.7%) (Table 2). This low proportion of HIV-infected participants with evidence of antiretroviral therapy reflects the fact that 45% of participants had missing data on antiretroviral treatment.

**Table 2:** Characteristics of people with HIV after weighting by province and age category.

Variable	All ages	Adults 15–49 years	Children 0–14 years
Unweighted number	331	270	71
Weighted percentage		90.6	9.4
Timing of HIV infection (%)			
Recent	2.7	1.0	3.3
Past	97.0	99.0	94.9
Missing data	0.3	NA	1.8
History of antiretroviral therapy (%)^a^			
Antiretroviral treatment	82.5	80.5	83.8
No antiretroviral treatment	17.5	19.5	16.2
CD4^+^ cell count in cells/μl (%)			
<100	4.8	7.0	1.8
100–200	7.6	7.7	3.6
200–350	19.3	27.8	3.6
350–500	23.6	28.6	13.1
≥500	41.7	28.1	73.1
Missing	3.0	0.8	4.8
Viral load (%)			
Viral suppression	39.6	49.2	35.7
No viral suppression	58.3	47.8	63.1
Missing	2.1	3.0	1.2

a Persons living with HIV missing self-reported antiretroviral treatment history and missing test results for biomarkers of ART (comprising 45% of PWH) were removed from the denominators.

### Measles seroprevalence by HIV infection status

**Fig. 1:**
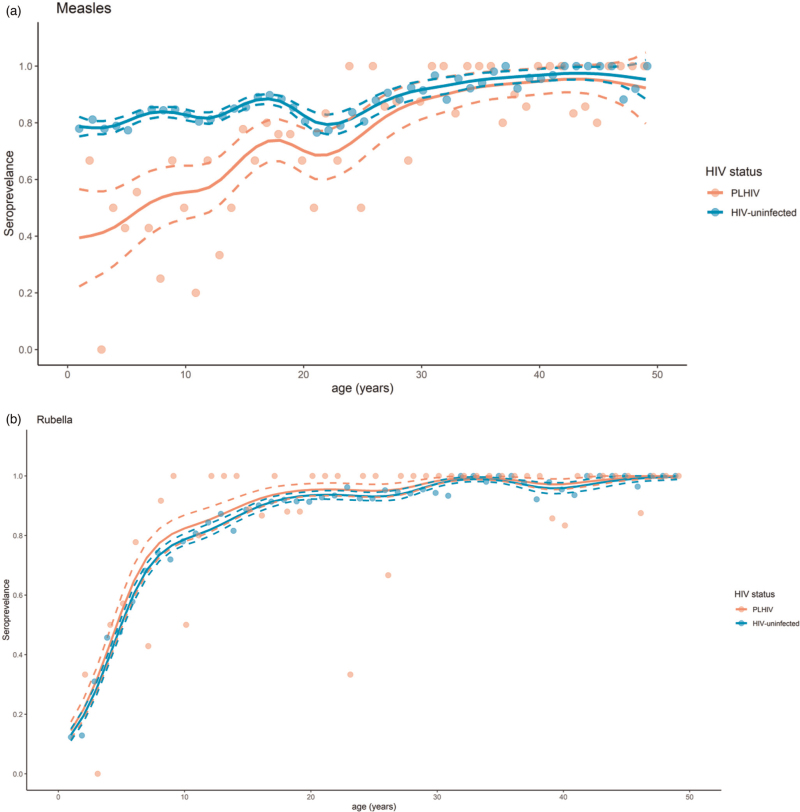
Measles and rubella age-specific seroprevalence.

Measles seroprevalence increased with age among both PWH and HIV uninfected participants but was lower among children, adolescents, and young adults living with HIV compared with HIV-uninfected participants younger than approximately 30 years of age based on the hierarchical generalized additive model (Fig. 1a, Table 3). Measles seroprevalence was only 47.2% [95% confidence interval (CI): 32.7, 61.7) among children younger than 10 years living with HIV compared to 76.4% (95% CI: 74.9, 78.0) among HIV-uninfected children in the same age category (Table 3). In the log-binomial regression analysis adjusting for HIV infection status, sex, and age category (younger than 9 years, 10−19 years, and 20−49 years), PWH were less likely to be measles seropositive than HIV-uninfected individuals across all age groups (Table 4, combined effects row), although the difference was greatest in children younger than 10 years for whom the adjusted prevalence ratio was 0.23 (95% CI: 0.13, 0.42). The adjusted prevalence ratio of 0.63 for those 20−49 years of age is influenced by the lower measles seroprevalence among those 20−30 years of age and does not capture the similar measles seroprevalence between adults living with HIV and those who were HIV uninfected older than 30 years. PWH who did not achieve viral load suppression were less likely to be measles seropositive than those who did (adjusted prevalence ratio 0.15, 95% CI: 0.06, 0.38), but no associations were observed in univariate analyses between measles seroprevalence and timing of HIV infection, antiretroviral treatment, and immunosuppression defined as CD4^+^ T-lymphocyte cell count below 200 cells/μl (Table S1, Supplemental Digital Content).

The lines represent generalized additive model fits mean (solid) and 95% confidence intervals (dashed). The data is grouped by age in years; year 0 includes only samples 9−11 months old. Rubella containing vaccine was not available in the public sector − prior to the serosurvey.

**Table 3:** Measles and rubella seroprevalence by age and HIV infection status.

	Seroprevalence among HIV-uninfected (95% CI)	Seroprevalence among PWH (95% CI)	*P*-value^∗^
Measles			
0–9 years	76.4% (74.9, 78.0)	47.2% (32.7, 61.7)	<0.001
10–19 years	84.9% (83.7, 86.1)	66.0% (55.6, 76.3)	<0.001
20–49 years	88.2% (85.8, 90.6)	85.6% (75.6, 95.5)	0.61
Rubella			
0–9 years	44.3% (42.6, 46.1)	68.6% (55.4, 81.8)	<0.001
10–19 years	86.4% (85.3, 87.6)	88.6% (81.0, 96.1)	0.58
20–49 years	95.4% (94.0, 96.9)	97.8% (95.9, 99.8)	0.56

PWH, people with HIV.

**Table 4:** Univariate and multivariate analyses of risk factors associated with measles and rubella seroprevalence among persons living with HIV and HIV-uninfected individuals.

		Measles seroprevalence ratio			Rubella seroprevalence ratio	
Variable	Measles seroprevalence (%)	Unadjusted prevalence ratio (95% CI)	Adjusted prevalence ratio (95% CI)	Rubella seroprevalence (%)	Unadjusted prevalence ratio (95% CI)	Adjusted prevalence ratio (95% CI)
HIV status						
HIV negative	84.5	ref	ref (0–9 years)	75.7	ref	ref (0–9 years)
HIV positive	81.5	0.9 (0.51, 1.58)	0.23 (0.13, 0.42)	95.1	6.87 (4.28, 11.01)	2.15 (1.26, 3.67)
Sex						
Female	85.5	ref	ref	75.3	ref	
Male	83.2	0.83 (0.7, 0.98)	0.84 (0.71, 1.01)	74.3	0.95 (0.84, 1.07)	
Setting						
Rural	84.2	ref		78.1	ref	ref
Urban	84.6	1.03 (0.87, 1.23)		72.4	1.37 (1.20, 1.56)	1.09 (0.95, 1.25)
Age category						
0–9 years	80.1	ref	ref (HIV negative)	44.6	ref	ref (HIV negative)
10–19 years	84.6	1.72 (1.51, 1.95)	1.37 (1.19, 1.56)	86.5	1.72 (1.51, 1.95)	7.47 (6.61, 8.45)
20–49 years	87.9	2.27 (1.78, 2.91)	1.82 (1.41, 2.34)	95.7	2.27 (1.78, 2.91)	24.52 (17.62, 34.14)
Combined effects::						
HIV status and age category						
HIV positive 0–9 years (ref = HIV negative 0–9 years)			0.23 (0.13, 0.42)			
HIV positive 10–19 years (ref = HIV negative 10–19 years)			0.77 (0.65, 0.86)			
HIV positive 20–49 years (ref = HIV negative 20–49 years)			0.63 (0.43, 0.80)			

### Rubella seroprevalence by HIV infection status

Also as expected, rubella seroprevalence increased with age among both PWH and HIV-uninfected participants, reflecting cumulative exposure to rubella virus in the absence of rubella vaccination (Fig. 1b, Table 3). Overall, rubella seroprevalence was higher among PWH (95.1%) than HIV-uninfected individuals (75.7%) (adjusted prevalence ratio: 2.15, 95% CI: 1.26, 3.67) (Table 4). Persons living in urban areas were more likely to be rubella seropositive than those living in rural areas in the univariate analysis but not in the adjusted model (adjusted prevalence ratio: 1.09, 95% CI: 0.95, 1.25). Rubella seroprevalence for children younger than 10 years was 68.6% (95% CI 55.4, 81.8) among children living with HIV and 44.3% (95% CI: 42.6, 46.1) among HIV-uninfected children (Table 3). After ten years of age, rubella seroprevalence was similar for PWH and HIV-uninfected individuals: 88.6% (95% CI: 81.0−96.1) among PWH 10–19 years and 86.4 (85.3%, 87.6%) for HIV-uninfected individuals, and 97.8% (95.9%, 99.8%) among PWH older than 20 years and 95.4% (94.0%, 96.9%) for HIV-uninfected individuals (Table 3).

Among PWH, rubella seroprevalence did not differ significantly between those living in rural (96.0%) or urban (93.8%) settings, those receiving or not receiving antiretroviral therapy, those with recent or past HIV infection, those with CD4^+^ T-lymphocyte count greater or less than 200 cells/μl, or those with or without viral load suppression (Table S1, Supplemental Digital Content). Only age was significantly associated with rubella seropositivity among PWH in the adjusted model (Table S1, Supplemental Digital Content).

## Discussion

This is the first nationally representative measles and rubella serosurvey for which the results were stratified by HIV infection status. Measles immunity gaps were identified among Zambian children, adolescents, and young adults living with HIV despite high measles vaccination coverage and access to antiretroviral therapy. Rubella seroprevalence, reflecting infection and not immunization, was higher among children living with HIV than uninfected children younger than 10 years of age, indicating greater exposure to rubella virus in this age group.

The WHO-UNICEF measles vaccine coverage estimates for Zambia in 2021 was 90% for MCV1 (down from 96% in 2020) and 81% for MCV2 (up from 74% in 2020) [21]. This high measles vaccine coverage is reflected in high measles seroprevalence estimates, although immunity gaps remain. We previously estimated measles seroprevalence in Zambia to be 82.8% (95% CI: 81.6, 83.9) for individuals younger than 50 years [23]. However, national estimates can mask heterogeneities in measles seroprevalence by location, age, and in specific subpopulations. Here we show that despite high measles vaccine coverage in Zambia, measles immunity gaps also exist in the population of children, adolescents and young adults living with HIV. We also show that although measles vaccination campaigns are held every fourth year in Zambia and providing every child at least two opportunities to catch-up by the age of 10 years, population immunity gaps still remain both among children living with HIV and HIV-uninfected children. One of the major reasons for these persistent population immunity gaps is failure of both routine and campaign activities to reach vulnerable populations. Lower measles seroprevalence among PWH could reflect lower measles vaccination coverage but vaccination history was not available to assess this hypothesis. In this setting lower measles seroprevalence most likely is due to higher rates of primary and secondary measles vaccine failure in children living with HIV, as has been shown in other studies summarized in systematic reviews [15–17].

According to UNAIDS, approximately 1.5 million people were living with HIV in Zambia in 2020, of whom 83 000 were children [22]. The proportion of PWH who were receiving antiretroviral therapy was 81% but was higher among adults (82%) than children (58%) [22]. The proportion of PWH receiving antiretroviral therapy in our study population was similar to the UNAIDS estimate but 45% of study participants had missing data on antiretroviral treatment. A large proportion of the missing data were from adolescents 10–19 years (41%) and adults 10–49 years (43%) (Figure S3, Supplemental Digital Content) Antiretroviral therapy does not restore measles immunity in children vaccinated prior to immune reconstitution [12] but prolongs survival [28]. Thus, increasing access to antiretroviral therapy leading to longer survival, combined with higher rates of primary and secondary measles vaccine failure among children living with HIV, explains the measles immunity gap among children, adolescents, and young adults living with HIV in Zambia. Interestingly, failure to achieve viral suppression was strongly associated with measles seronegativity, consistent with a study of Kenyan children living with HIV and receiving antiretroviral therapy in whom seroconversion after measles vaccination was positively associated with HIV viral load suppression [13].

The association between rubella seroprevalence and HIV infection status was different than for measles, with rubella seropositivity higher among children living with HIV than in HIV uninfected children, but similar in older age groups. This difference is because the serosurvey was conducted before the introduction of rubella vaccine in Zambia and thus rubella seroprevalence reflects infection with rubella virus. The higher rubella seroprevalence among children living with HIV may be due to greater exposure to rubella virus in urban settings, as demographic factors such as higher birth rates, increased mobility, and larger population size facilitate rubella virus transmission [29]. A study in rural southern Zambian found lower rubella seroprevalence among children 5–15 years old living with HIV compared to uninfected youth [30].

This study had several limitations, most arising from the fact that this was a secondary analysis of specimens in a biorepository collected for another purpose. The original ZAMPHIA survey did not collect information on measles and rubella vaccination status and thus we were unable to assess associations between measles and rubella vaccination and seroprevalence at the individual level, nor could we distinguish vaccine failure from failure to vaccinate. The number of children living with HIV was small, limiting our ability to draw inferences regarding risk factors associated with measles and rubella seroprevalence in this age group. We were unable, for example, to assess whether initiation of antiretroviral therapy before measles vaccination was more likely to result in measles seropositivity after vaccination.

### Conclusion

These findings highlight the need to implement the WHO recommendation to revaccinate children living with HIV against measles after achieving immune reconstitution with antiretroviral therapy. Consideration also should be given to vaccinating adolescents and young adults living with HIV in communities at risk of measles outbreaks. Policies should be adapted to facilitate tailored strategies to vaccinate older individuals and those in need of revaccination after immune reconstitution.

## Acknowledgements

We thank the participants who provided specimens for the ZAMPHIA biorepository. We would also like to thank Lameck Chirwa of the University of Zambia, the CDC Zambia country office, and the Zambia Ministry of Health Directorate of Public Health and Research for authorizing access to the ZAMPHIA biorepository. We thank our colleagues Evans Betha, Samson Mwale, and Shepherd Nehanda at TDRC and Nosiku Sitali, Meikess Kabwe, and Kwalela Kwalela at UNZA for conducting the laboratory testing.

Funding: The Bill and Melinda Gates Foundation (Grant # OPP1094816)

Author contributions: Conceptualization: S.M., K.H., A.C. and W.J.M.; data collection: S.M., F.D.M., W.J.M., I.C., I.M., G.M., M.M. and K.H., H.N., L.M., G.C.; methodology: S.M., Y.Y., K.H., W.J.M. and A.K.W.; software and analysis: S.M., Y.Y., K.H., A.C. and A.K.W.; writing—original draft preparation: S.M., Y.Y., K.H. and A.K.W.; writing—review and editing: W.J.M., K.H., A.C., A.K.W., F.D.M., I.C., I.M., G.C., M.M., G.M., H.N., L.M., S.M.; visualization: Y.Y. and A.W.; supervision: S.M. and W.J.M.; funding acquisition: W.J.M. and K.H. All other authors critically reviewed results. All authors have read and agreed to the published version of the manuscript. W.M. is responsible for the overall content as a guarantor.

Data sharing: Data dictionary will be made available with publication as supplemental material. Study protocol can be made available upon request to the corresponding author (smutemb1@jhmi.edu) following publication.

### Conflicts of interest

There are no conflicts of interest.

## Supplementary Material

Supplemental Digital Content

## References

[1] Minta AA Ferrari M Antoni S Portnoy A Sbarra A Lambert B. Progress toward regional measles elimination - worldwide, 2000−2021. *MMWR Morb Mortal Wkly Rep* 2022; 71:1489–1495. 36417303 10.15585/mmwr.mm7147a1 PMC9707362

[2] Dixon MG Ferrari M Antoni S Li X Portnoy A Lambert B. Progress toward regional measles elimination - worldwide, 2000–2020. *MMWR Morb Mortal Wkly Rep* 2021; 70:1563–1569. 34758014 10.15585/mmwr.mm7045a1 PMC8580203

[3] Shet A Carr K Danovaro-Holliday MC Sodha SV Prosperi C Wunderlich J. Impact of the SARS-CoV-2 pandemic on routine immunisation services: evidence of disruption and recovery from 170 countries and territories. *Lancet Glob Health* 2022; 10:e186–e194. 34951973 10.1016/S2214-109X(21)00512-X PMC8691849

[4] Ho LL Gurung S Mirza I Nicolas HD Steulet Claudia Burman AL. Impact of the SARS-CoV-2 pandemic on vaccine-preventable disease campaigns. *Int J Infect Dis* 2022; 119:201–209. 35398300 10.1016/j.ijid.2022.04.005 PMC8985404

[5] World Health Organization. Rubella vaccines: WHO position paper. July 2020. Wkly Epidemiol Rec 2020;95:27.

[6] Wariri O Nkereuwem E Erondu NA Edem B Nkereuwem OO Idoko OT. A scorecard of progress towards measles elimination in 15 west African countries, 2001−19: a retrospective, multicountry analysis of national immunisation coverage and surveillance data. *Lancet Glob Health* 2021; 9:e280–e290. 33607028 10.1016/S2214-109X(20)30481-2 PMC7900524

[7] Truelove SA Graham M Moss WJ Metcalf CJE Ferrari MJ Lessler J. Characterizing the impact of spatial clustering of susceptibility for measles elimination. *Vaccine* 2019; 37:732–741. 30579756 10.1016/j.vaccine.2018.12.012 PMC6348711

[8] Moss WJ Shendale S Lindstrand A O’Brien KL Turner N. Feasibility assessment of measles and rubella eradication. *Vaccine* 2021; 39:3544–3559. 34045102 10.1016/j.vaccine.2021.04.027

[9] Scott S Mossong J Moss WJ Cutts FT Cousens S. Predicted impact of the HIV-1 epidemic on measles in developing countries: results from a dynamic age-structured model. *Int J Epidemiol* 2008; 37:356–367. 18234739 10.1093/ije/dyn007

[10] Scott S Moss WJ Cousens S Beeler JA Audet SA Mugala N. The influence of HIV-1 exposure and infection on levels of passively acquired antibodies to measles virus in Zambian infants. *Clin Infect Dis* 2007; 45:1417–1424. 17990222 10.1086/522989

[11] Moss WJ Scott S Mugala N Mugala N Ndhlovu Z Beeler JA. Immunogenicity of standard-titer measles vaccine in HIV-1-infected and uninfected Zambian children: an observational study. *J infect Dis* 2007; 196:347–355. 17597448 10.1086/519169

[12] Rainwater-Lovett K Nkamba HC Mubiana-Mbewe M Bolton-Moore C Moss WJ. Changes in measles serostatus among HIV-infected Zambian children initiating antiretroviral therapy before and after the 2010 measles outbreak and supplemental immunization activities. *J infect Dis* 2013; 208:1747–1755. 23911708 10.1093/infdis/jit404 PMC3814842

[13] Newman LP Njoroge A Magaret A Chohan BH Gitomea VW Wald A. Sustained responses to measles revaccination at 24 months in HIV-infected children on antiretroviral therapy in Kenya. *Pediatr Infect Dis J* 2017; 36:1148–1155. 28198789 10.1097/INF.0000000000001572 PMC5554743

[14] World Health Organization. **Measles vaccines: WHO position paper**. April 2017. *Wkly Epidemiol Rec* 2017;**92**:205−227.

[15] Scott P Moss WJ Gilani Z Low N. Measles vaccination in HIV-infected children: systematic review and meta-analysis of safety and immunogenicity. *J infect Dis* 2011; 204:S164–S178. 21666158 10.1093/infdis/jir071

[16] Mutsaerts E Nunes MC van Rijswijk MN Klipstein-Grobusch K Grobbee DE Madhi SA. Safety and immunogenicity of measles vaccination in HIV-infected and HIV-exposed uninfected children: a systematic review and meta-analysis. *EClinicalMedicine* 2018; 1:28–42. 31193646 10.1016/j.eclinm.2018.06.002 PMC6537570

[17] Mehtani NJ Rosman L Moss WJ. Immunogenicity and safety of measles vaccine in HIV-infected children: an updated systematic review. *Am J Epidemiol* 2019; 188:2240–2251. 31210268 10.1093/aje/kwz144

[18] Loevinsohn G Rosman L Moss WJ. Measles seroprevalence and vaccine responses in human immunodeficiency virus-infected adolescents and adults: a systematic review. *Clin Infect Dis* 2019; 69:836–844. 30452621 10.1093/cid/ciy980

[19] Rainwater-Lovett K Moss WJ. Immunologic basis for revaccination of HIV-infected children receiving HAART. *Future Virol* 2011; 6:59–71. 21339832 10.2217/fvl.10.75 PMC3039418

[20] Crum NF Ahmad A. Immunity against measles in people with HIV: the need for more research and surveillance. *AIDS* 2022; 36:1305–1306. 35833681 10.1097/QAD.0000000000003254

[21] World Health Organization. Immunization Zambia 2022 country profile. World Health Organization. 6 July, 2022. Available at: https://www.who.int/publications/m/item/immunization-zmb-2022-country-profile [Accessed July 23, 2022].

[22] UNAIDS. Zambia country factsheet. UNAIDS, 2020. Available at: https://www.unaids.org/en/regionscountries/countries/zambia [Accessed July 23, 2022].

[23] Carcelen AC Amy WK Moss WJ Chilumba I Mutale I Chongwe G. Leveraging a national biorepository in Zambia to assess measles and rubella immunity gaps across age and space. *Sci Rep* 2022; 12:10217. 35715547 10.1038/s41598-022-14493-3 PMC9204687

[24] Zambia Ministry of Health. Zambia Population-Based HIV Impact Assessment (ZAMPHIA). Zambia Ministry of Health, 1 Jan, 2019. Available at: https://phia.icap.columbia.edu/wp-content/uploads/2019/02/ZAMPHIA_Summary_Sheet_Final.pdf [Accessed January 17, 2022].

[25] Wood S. *Generalized additive models: an introduction with R*. 2nd ed. Boca Raton, Florida: Chapman and Hall/CRC; 2017.

[26] Lumley T. Analysis of complex survey samples. *J Stat Soft* 2004. 9.

[27] Wickham H. Ggplot2: elegant graphics for data analysis. 2nd ed New York: Springer; 2016.

[28] Sutcliffe CG van Dijk JH Bolton C Persaud D Moss WJ. Effectiveness of antiretroviral therapy among HIV-infected children in sub-Saharan Africa. *Lancet Infect Dis* 2008; 8:477–489. 18652994 10.1016/S1473-3099(08)70180-4

[29] Winter AK Moss WJ. Rubella. *Lancet* 2022; 399:1336–1346. 35367004 10.1016/S0140-6736(21)02691-X

[30] Sutcliffe CG Searle K Matakala HK. Measles and rubella seroprevalence among HIV-infected and uninfected Zambian youth. *Pediatr Infect Dis J* 2017; 36:301–306. 27879554 10.1097/INF.0000000000001422 PMC5303148

