# Supplementary material for: Measles immunity gaps among children and adolescents with HIV in Zambia despite high measles vaccination and antiretroviral therapy coverage
Source: AIDS. 2023 Jun 28;37(13):2021–9. doi: 10.1097/QAD.0000000000003634 (PMC10664789; doi:10.1097/QAD.0000000000003634)

Measles Immunity Gaps Among Children and Adolescents with HIV in Zambia Despite High Measles Vaccination and Antiretroviral Therapy Coverage

Figure S1. Study flow diagram.

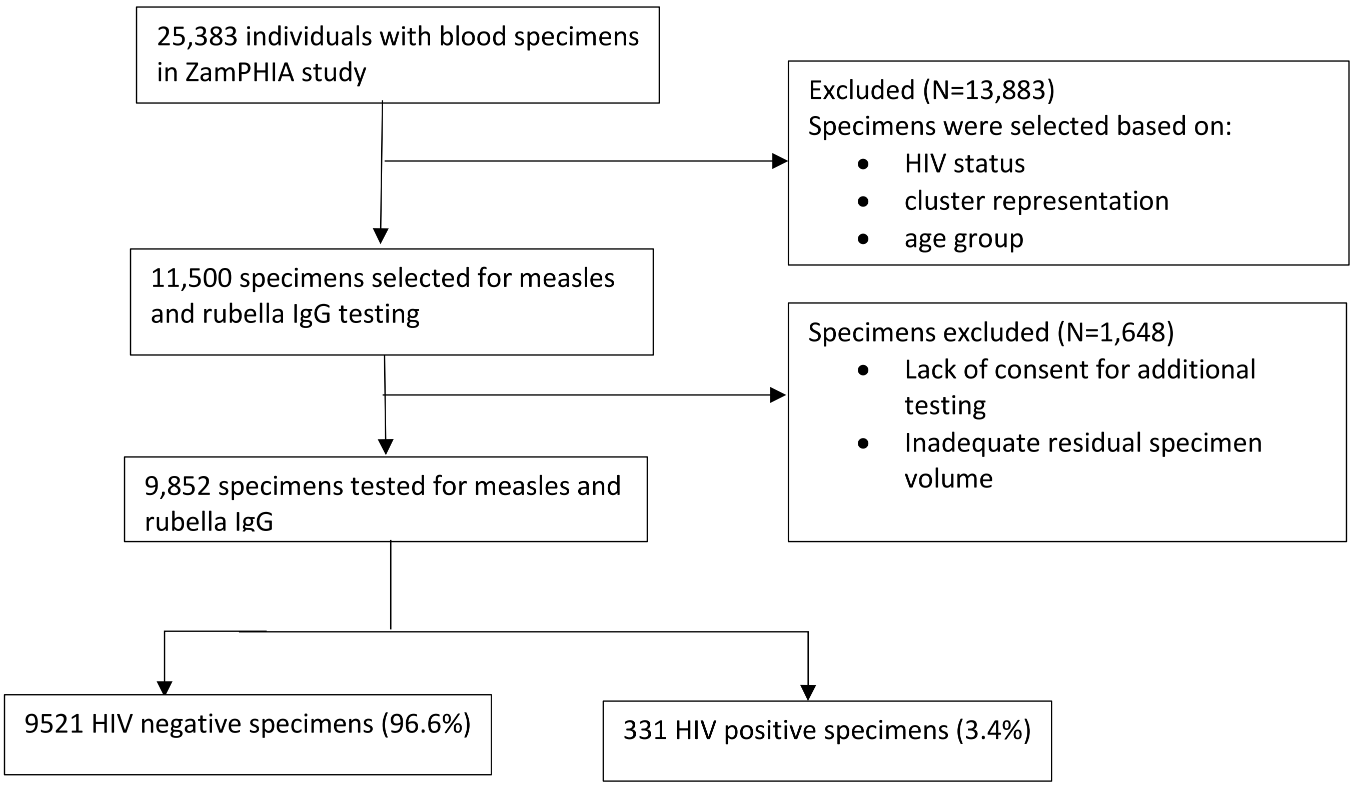

Table S1: Univariate and multivariate results showing risk factors associated with measles and rubella seroprevalence among people living with HIV

Variable	Measles Seroprevalence (%)	Measles Seroprevalence Ratio		Rubella Seroprevalence (%)	Rubella Seroprevalence Ratio	
		Unadjusted Prevalence Ratio (95% CI)	Adjusted Prevalence Ratio (95% CI)		Unadjusted Prevalence Ratio (95% CI)	Unadjusted Prevalence Ratio (95% CI)
Sex						
Female	79.4	ref		96.3	ref	
Male	84.7	1.51			0.49	
		(0.6, 3.82)		93.1	(0.18, 1.37)	
Setting						
Rural	87.3	ref		96.0	ref	
Urban	77.1	0.52			1.35	
		(0.2, 1.35)		93.8	(0.50, 3.69)	
Age category						
0-9 years	46.2	ref	ref	68.6	ref	ref

10-19 years	67.0	2.21 (1.02, 4.82)	2.11 (0.87, 5.09)	88.6	3.42 (1.23, 9.52)	3.59 (1.32, 9.79)
20-49 years	85.6	6.51 (2.35, 18.01)	5.35 (1.91, 14.95)	97.8	21.02 (6.33, 69.8)	25.74 (8.29, 79.89)
HIV treatment						
On treatment	88.1	ref		95.2	ref	
Not on treatment	89.4	1.77 (0.49, 6.33)		95.1	0.87 (0.32, 2.37)	
**Timing of HIV infection (%)**						
Recent	83.0	ref		90.6	ref	
Past	81.4	0.92 (0.09, 8.93)		95.3	0.98 (0.11, 9.11)	
***CD4 count category* in cells/mm^3**						
<200	89.3	ref		94.1	ref	
200+	82.2	0.50		95.3	2.11	

		(0.17, 1.49)			(0.40, 11.23)	
Viral load suppression						
Suppressed	94.0	ref	ref	94.2	ref	ref
Not suppressed	68.2	0.13	0.15		1.48	2.45
		(0.05, 0.32)	(0.06, 0.38)	95.9	(0.56, 3.88)	(0.95, 8.68)

Figure S2. Rubella age-specific seroprevalence among rural and urban population. The lines represent generalized additive model fits mean (solid) and 95% confidence intervals (dashed). Red lines and dots represent the PLHIV, green lines and dots represent HIV-uninfected population. The data is grouped by age in years; year 0 includes only samples 9-11 months old. Rubella containing vaccine was not available in the public sector prior to the serosurvey.

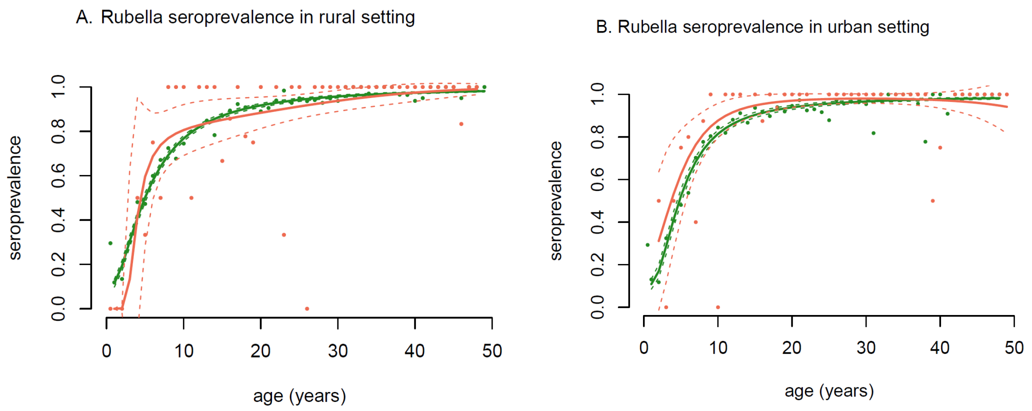

Figure S3. ART definition and information. The lab test was conducted for the biomarker in blood of first line ART treatments in Zambia at the time of this study (Efavirenz, Atazanavir, Lopinavir, or Nevirapine).

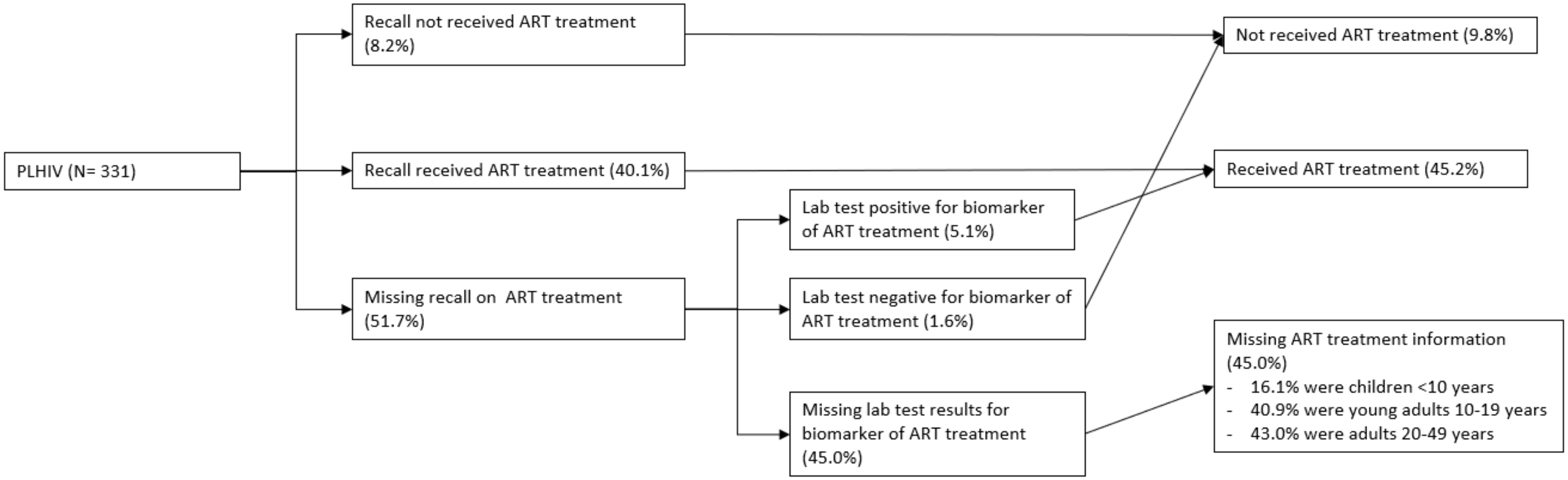

Supplement: Supplemental Digital Content [file aids-37-2021-s001.pdf]
